# Whole Genome Sequence of *Alternaria alternata*, the Causal Agent of Black Spot of Kiwifruit

**DOI:** 10.3389/fmicb.2021.713462

**Published:** 2021-09-20

**Authors:** Ke Huang, Jianming Tang, Yong Zou, Xiangcheng Sun, Jianbin Lan, Wei Wang, Panpan Xu, Xiangwei Wu, Rui Ma, Qi Wang, Zhenshuo Wang, Jia Liu

**Affiliations:** ^1^College of Landscape Architecture and Life Science, Institute of Special Plants, Chongqing University of Arts and Sciences, Chongqing, China; ^2^Institute of Microbial Ecology, Chongqing University of Arts and Sciences, Chongqing, China; ^3^College of Life Sciences, Northwest A&F University, Yangling, China; ^4^West China Biopharm Research Institute, West China Hospital, Sichuan University, Chengdu, China; ^5^QianTang Biotech Co. Ltd., Suzhou, China; ^6^Department of Plant Pathology, MOA Key Lab of Pest Monitoring and Green Management, College of Plant Protection, China Agricultural University, Beijing, China

**Keywords:** *Alternaria alternata*, black spot, genome, kiwifruit, mycotoxin

## Abstract

*Alternaria alternata* is a pathogen in a wide range of agriculture crops and causes significant economic losses. A strain of *A. alternata* (Y784-BC03) was isolated and identified from “Hongyang” kiwifruit and demonstrated to cause black spot infections on fruits. The genome sequence of Y784-BC03 was obtained using Nanopore MinION technology. The assembled genome is composed of 33,869,130bp (32.30Mb) comprising 10 chromosomes and 11,954 genes. A total of 2,180 virulence factors were predicted to be present in the obtained genome sequence. The virulence factors comprised genes encoding secondary metabolites, including non-host-specific toxins, cell wall-degrading enzymes, and major transcriptional regulators. The predicted gene clusters encoding genes for the biosynthesis and export of secondary metabolites in the genome of Y784-BC03 were associated with non-host-specific toxins, including cercosporin, dothistromin, and versicolorin B. Major transcriptional regulators of different mycotoxin biosynthesis pathways were identified, including the transcriptional regulators, polyketide synthase, P450 monooxygenase, and major facilitator superfamily transporters.

## Introduction

Kiwifruit (*Actinidia chinensis*) is a popular fruit worldwide. “Hongyang” is an excellent variety of red-flesh kiwifruit that is widely grown in China. Although the farmers employ a management system to limit fungal pathogens, black spot of kiwifruit often occurs during kiwifruit development. *A. alternata* is a saprophytic fungus in the order Hyphomycetes, Fungi Imperfecti. It has the ability to inhabit a wide range of plant an animal hosts. *A. alternata* is a saprophytic pathogen in fruits and is the causal agent of Alternaria rot in apple, pear, strawberry, melon, persimmon, and Brassica species (Nishimura and Kohmoto, 1983; Li et al., 2013; Meena and Samal, 2019). *A. alternata* has also been reported to be the causal agent of black rot in kiwifruit at both pre- and postharvest stages (Kwon et al., 2011).

*Alternaria alternata*, as well as other pathogenic fungi, exhibits an annual disease cycle that extends from one growing season to the next. The mycelia of *A. alternata* overwinters in its host and proliferates the following season through spore production. Moderate temperatures around 25°C (Lee et al., 2015; Zhu and Xiao, 2015) and high relative humidity over 96% (Lee et al., 2015) support *A. alternata* spore germination. Management practices, cultivar selection, and general cultural practices all play a role in the proliferation of *A. alternata* (Troncoso-Rojas and Tiznado-Hernández, 2014). Under suitable conditions, spores are released from overwintering plant debris and establish new infections in current year plant tissues. Germ tubes and hyphae develop once spores come into contact with the cuticle of host tissues (Troncoso-Rojas and Tiznado-Hernández, 2014). New conidia are produced in the newly infected host and are eventually released to re-infect the host.

In the present study, a strain of *A. alternata* (Y784-BC03) was isolated from “Hongyang” kiwifruit grown in Yongchuan District, Chongqing, China (29.3561°N, 105.9274°E). Its identification was confirmed by ITS gene sequence analysis. Koch postulates were also used to confirm that this *A. alternata* strain could infect and cause black spot in kiwifruit. A whole genome sequence of *A. alternata* (Y784-BC03) was obtained for further studies of isolate differentiation and to develop a better understanding of pathogenicity and virulence in this fungal pathogen.

## Materials and Methods

### Isolation and Identification of the Causal Agent of Black Spot of Kiwifruit

Kiwifruits with symptoms of block spot were surface sterilized with 75% ethanol and then air-dried. A portion of the epidermis and underlying tissue of a region of the fruit with black spot symptoms was then collected. The surface-sterilized black spot tissues were then placed and cultured on potato dextrose agar (PDA) media. Colonies of the isolate were then purified using monosporic culture methods and subjected to genetic analysis. DNA was extracted from a purified colony and used to amplify the ITS gene using the primer pair, ITS1 TCCGTAGGTGAACCTGCGG and ITS4 TCCTCCGCTTATTGA-TATGC. The obtained sequences were used to calculate phylogenetic distance using Clustal X2 software program and MEGA 5.0 program. The isolate was identified as *A. alternata* (Y784-BC03) and further confirmed by genome sequencing. A whole genome sequence of the isolate was obtained using Oxford Nanopore sequencing technology (Lu et al., 2016).

### Pathogenicity of *A. alternata* (Y784-BC03)

A monosporic-derived culture was grown on PDA medium at 25°C for 5days. Petri plates were flooded with sterile distilled water, and the cultures gently brushed with a sterile glass rod to loosen spores. The spores were collected, and a spore count was determined under a microscope with the aid of a hemocytometer. The spore suspensions were adjusted to 10^4^, 10^5^, 10^6^, and 10^7^ spores/ml. Healthy kiwifruits were used to assay the pathogenicity of the obtained isolate. Fruits were surface sterilized with 75% ethanol, and three small wounds (10mm deep) were distributed on three sides of each fruit. Every wound was inoculated with 10μl of one the prepared spore suspensions. The inoculated fruits were placed at 25°C and observed every day for evidence of infections. Wounds inoculated with sterilized water or a biocontrol yeast (*Candida diversa*) adjusted 10^4^ spores/ml (Liu et al., 2017) were used as a control. Every treatment included 15 fruits, and the assay was repeated three times. The fruits were cut in half in order to observe disease symptoms to determine whether the Y784-BC03 strain of *A. alternata* was the causative agent of black rot decay. The percentage of disease incidence was determined using the following calculation: IC=(*n*/*N*)×100; where IC=incidence; *n*=number of lesion spots of kiwifruits; and *N*=the total number of wounds.

### Genome Sequencing

Strain Y784-BC03 was grown on PDA medium at 25°C for 5days and at 20°C for 7days. Genomic DNA was extracted from harvested mycelia using a DNeasy Blood & Tissue Kit (Qiagen, Hilden, Germany) according to the manufacturer’s instructions. Genome sequencing of Y784-BC03 was conducted using Nanopore PromethION technology (Ox-ford Nanopore, Oxford, United Kingdom) using size-selected (30–80kb) DNA prepared with BluePippin (Sage Science, Beverly, MA, United States). Guppy software (version 3.2.0, Oxford Nanopore Technologies-ONT, Oxford, United Kingdom) was used for base calling of the raw signal data. The obtained sequences were then filtered to remove short reads (<5kb) and reads with low-quality bases and/or containing adapter sequences. An Illumina Navoseq6000 platform (Illumina, CA, United States) was also used to produce 200bp pair reads. FastQC (Version 0.11.9)^1^ and trimmomatic-0.38 (Bolger et al., 2014) with default parameters were used to filter the resulting Illumina reads. Filtered Illumina reads were than further processed using racon pipelines and pilon software (Version 1.22; Walker et al., 2014), to improve the genome assembly.

### Genome Assembly and Annotation

The genomic sequence data from the Nanopore platform were subjected to an iterative process and assembled in the following steps. First, ONT software was used to obtain high-quality long sequencing reads. The Canu model of ONT (Koren et al., 2017) based on inductive statistics was used to calculate the overlap of data and select similar sequences to decrease noisy reads. Second, the filtered data were then assembled using NECAT^2^ software, based on splicing of overlaps. Third, BUSCO (Seppey et al., 2019) analysis was used to conduct a quantitative assessment of the completeness of the genome assembly and annotation.

RepeatMasker and RepeatProteinMask (v.3.3.0)^3^ were used to predict known repetitive sequences. Augustus^4^ (Stanke et al., 2004), GeneMark^5^ and Stanford Network Analysis Project (SNAP)^6^ were used to predict genome structure based on the characteristics of the sequence statistics. Genome sequence data of *A. alternata* FERA_1177 (GCA_004154755.1), *A. alternata* SRC1lrK2f (GCA_001642055.1), and *A. alternata* Z7 (GCA_001572055.1) were used for the prediction of homologs, and the results were integrated using EVidenceModeler^7^ (Haas et al., 2008). Genes were functionally annotated by BLAST searches against the NR,^8^ NT,^9^ PFAM^10^ (Finn et al., 2014), egg-NOG^11^ (Powell et al., 2012), GO^12^ (Ashburner et al., 2000), and KEGG^13^ (Kanehisa et al., 2012) databases.

BLAST searches of the known non-coding RNA, non-coding RNA containing rRNA, snRNA, and miRNA were conducted using the Rfam^14^ database (Kalvari et al., 2018), and RNAscan-SE^15^ (Lowe and Chan, 2016) was used to predict tRNA.

### Genome Phylogeny and Collinearity Analysis

OrthoMCL^16^ was used to explore single-copy homologous proteins by retrieving the protein data of eight genomes, including *A. alternata* (Y784-BC03), *A. alternata* SRC1lrK2f (GCA_001642055.1) isolated from effluent of coal mine drainage (Zeiner et al., 2016), *A. alternata* Z7 (GCA_001572055.1) isolated from tangerine (Wang et al., 2019), *A. tenuissima* FERA_1177 (GCA_004154755.1) and *A. tenuissima* FERA 1164 (GCA_004156015.1) from apple, *A. tenuissima* FERA 23450 (GCA_004154735.1), *A. arborescens* RGR 97.0013 (GCA_004155955.1), and *A. arborescens* strain RGR 97.0016 (GCA_004154815.1; Supplementary Table S1; Armitage et al., 2020). Single-copy orthologous genes shared by all eight genomes were further aligned using MUSCLE (version 3.8.31; Edgar, 2010). Gblocks (version 0.91b; Talavera and Castresana, 2007) was then used to eliminate poorly aligned positions and divergent regions using the default parameter settings. Phylogenetic analysis was implemented using RAxML^17^ (Stamatakis, 2014) based on the maximum likelihood method, and the accuracy of the tree topology was assessed by bootstrap analysis with 1,000 re-sampling replicates. *A. arborescens* RGR 97.0013 (GCA_004155955.1), *A. alternata* SRC1lrK2f (GCA_001642055.1), and the assembled genome of Y784-BC03 were subjected to a collinearity analysis to assess the homology between related chromosomes.

### Identification of Virulence Factors in the Genomes

The protein sequence of the fungal virulence factors in the virulence factor database (Liu et al., 2019) was used to construct a local virulence factor library, which was aligned with the annotated genome-wide data to identify and obtain the information on the most important virulence factors and more important virulence factors in *A. alternata*. The predicted virulence factors were identified based on previous reports in the literature (Bradshaw and Zhang, 2006; Schwelm and Bradshaw, 2010; Roze et al., 2013; Wight et al., 2013; Lee et al., 2015; Gunasinghe et al., 2016; Tsuge et al., 2016; Ren et al., 2017; Muñoz et al., 2018; Meena and Samal, 2019; Gil-Serna et al., 2020). The number of virulence factors was rather small, containing only 2,180 coded genes (Supplementary Table S8). Therefore, we were able to artificially classify the virulence factors. The classified virulence factors were then located on chromosomes based on the constructed *A. alternata* (Y784-BC03) annotation database using R-4.03.

## Results

### Pathogen Isolation and Identification

**Figure 1:**
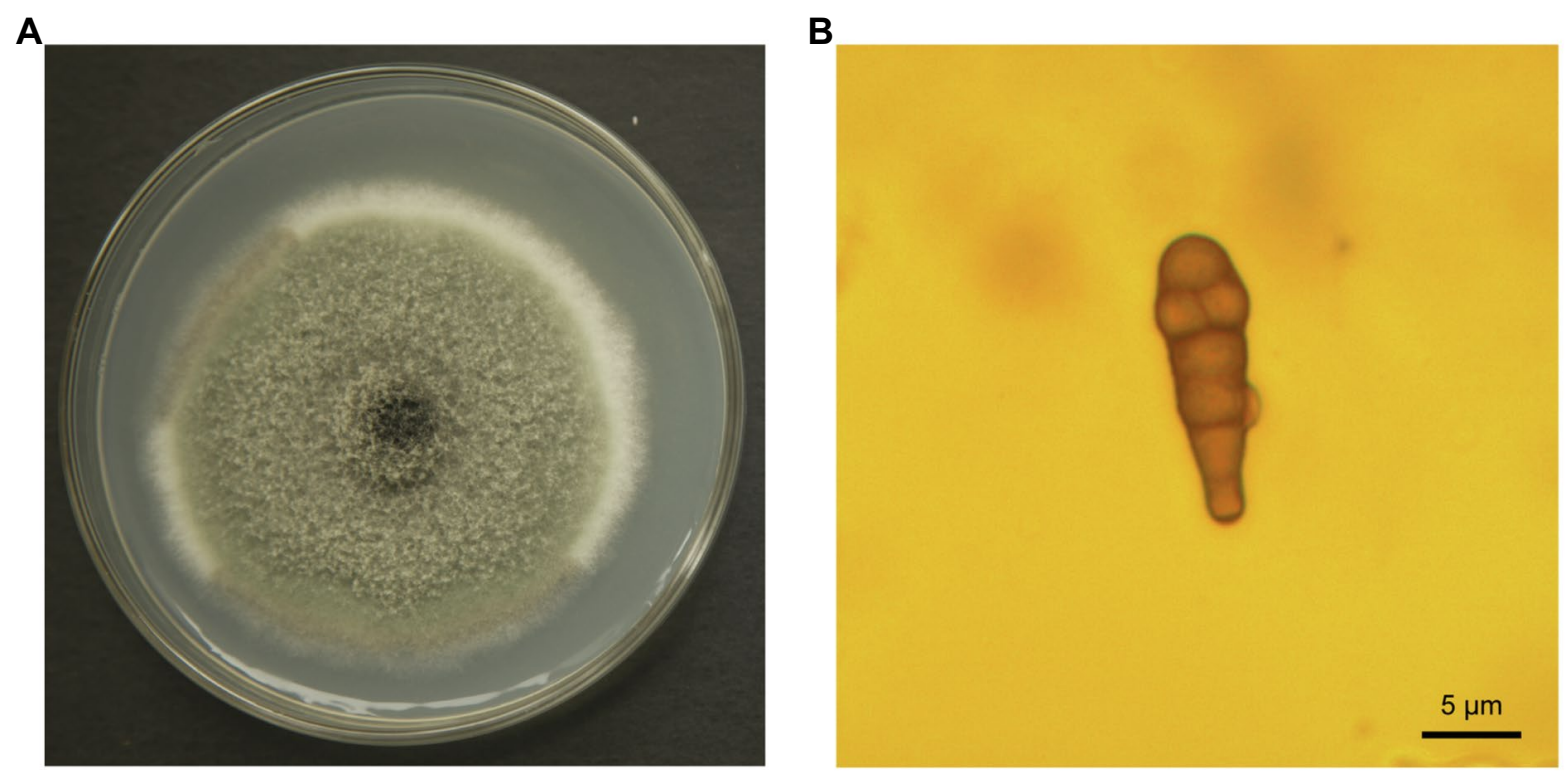
*Alternaria alternata* (Y784-BC03) morphology. Colony of *A. alternata* growing on potato dextrose agar **(A)** after 4days of culture. Conidia of *A. alternata* (Y784-BC03) at a magnification of ×100 **(B)**.

**Figure 2:**
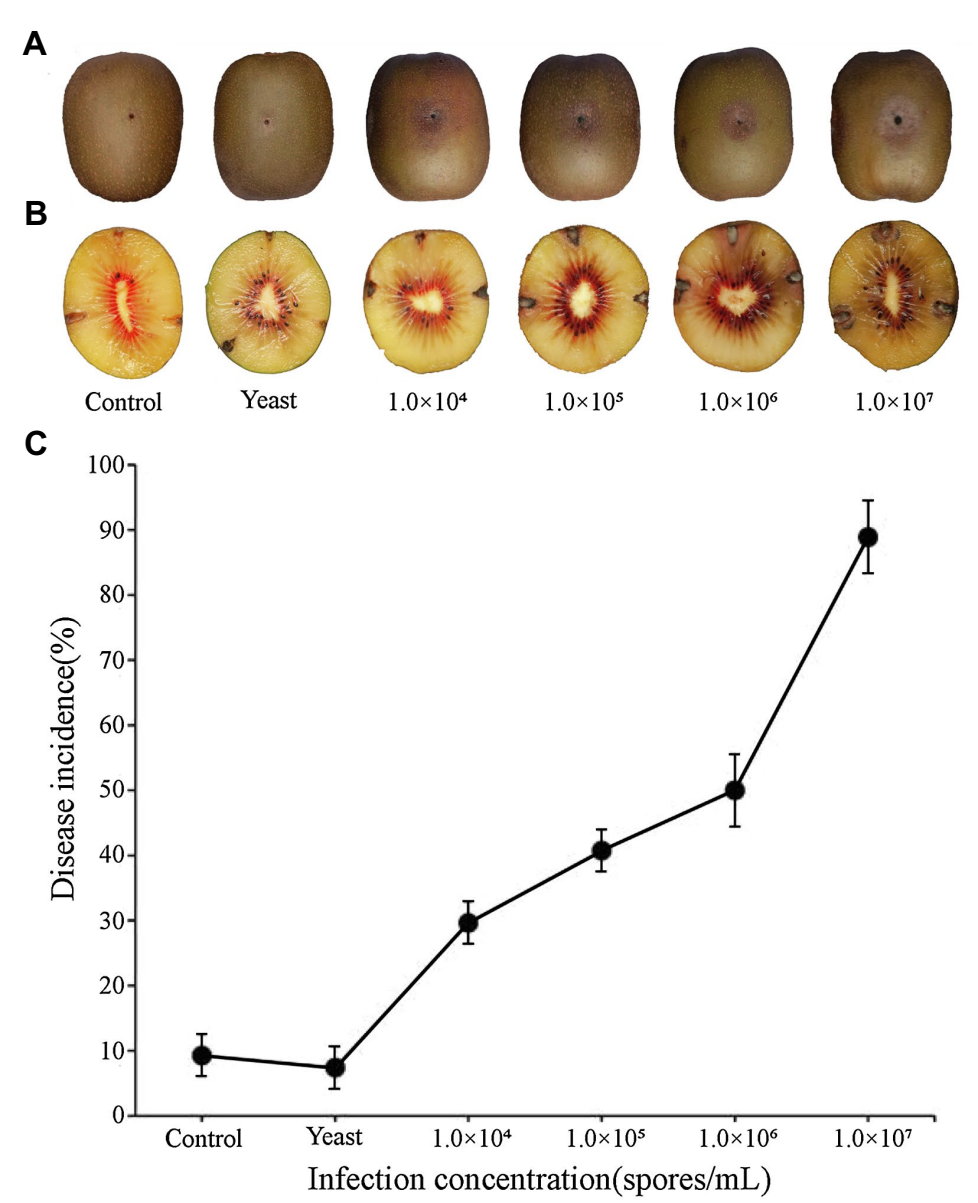
Black spots symptoms on kiwifruit inoculated with different spore concentrations of *A. alternata* (Y784-BC03). Kiwifruit black spots after 4days as a result of the inoculation of wounds with different concentrations of *A. alternata* (Y784-BC03) **(A)**. Appearance of mycelia in kiwifruit black spot infection sites **(B)**. *A. alternata* (Y784-BC03) disease incidence associated with the inoculation of wounded fruit with different concentrations of spores **(C)**.

Epidermal tissues of kiwifruit with the appearance of black rot were removed and cultured on PDA media. Twelve isolates were obtained from the colony and purified in culture. One strain, identified as Y784-BC03, was purified and grown from a monosporic culture. The Y784-BC03 mycelia appeared gray after 4days of culture at 25°C (Figure 1A). The conidia of Y784-BC03 were ovate in shape, and individual conidia were divided by transverse and vertical walls (Figure 1B), which is the typical morphology of *A. alternata* conidia (Kwon et al., 2011). Wounded fruits were inoculated with the prepared suspensions to determine the pathogenicity of the Y784-BC03 isolate. After 4days, black spots appeared on fruits that had been inoculated with 10^7^ spores/ml. Over time, the black spots enlarged and the underlying fruit tissues became soft and decayed (Figure 2A).

Fungal mycelia were also observed in central portion of the black spots at 10days post-inoculation (Figure 2B). Samples of the mycelia were retrieved and identified as *A. alternata*. As shown in Figure 2C, disease incidence significantly increased (*p*<0.01) with the increase in the spore concentration used to inoculate the fruit. Disease incidence was 29.62% at 10^4^ spores/ml, while, and 88.89% at 10^7^ spores/ml. Incidences of any infections were significantly lower (*p*<0.01) in control fruit inoculated with sterile water or yeast than they were in fruit inoculated with *A. alternata*. Notably, several of the control wounds (water or yeast) healed naturally (Figure 2A).

### Genome Assembly

A genome sequence and assembly of *A. alternata* (Y784-BC03) was constructed using a long-sequencing Nanopore, a short-read Illumina platforms. A total of 21,279,340,610bp were acquired. The whole genome of Y784-BC03 was determined to comprise 33,869,130bp (32.30Mb), which is similar to the genome size of other previously reported *A. alternata* genomes (Dang et al., 2015; Bihon et al., 2016; Nguyen et al., 2016; Gebru et al., 2020). The mapping rate between reads and assembled sequences was 99.50%.

The sequencing depth was 266X, and the coverage of the genome was 99.97% (Samtools depth). The structure of the Y784-BC03 genome was very similar to the genome of *A. solani* (Wolters et al., 2018). The final genome assembly comprises 12 contigs with an N50 value of 3,075,098bp (3.075m), which was improved from 3,068,336bp (3.068m) after additional refinement. Among the12 contigs, the 10 largest contigs were defined as chro-mosome1 (ch1) to Chr10 (Table 1). Among the assigned 10 chromosomes, 8 chromosomes (Chr01, Chr02, Chr03, Chr04, Chr04, Chr08, Chr09, and Chr10) were telomeric repeats (TTAGGG) on only one of the ends. Two others, Chr05 and Chr07, were larger than 10M and missing telomeric repeats at the ends. The remaining contigs 11 and contigs12 were too small to assemble into the genome structure and annotations of genes could not be accomplished.

**Table 1:** Summary of genome assembly statistics for *A. alternata* (Y784-BC03).

Chromosome	Length (bp)	Coverage	GC content (%)
Chr01	6,758,422	264×	51.23
Chr02	5,558,083	267×	51.03
Chr03	3,517,007	263×	50.99
Chr04	3,075,098	262×	50.88
Chr05	2,923,236	262×	50.91
Chr06	2,603,968	262×	50.80
Chr07	2,544,088	289×	51.02
Chr08	2,480,139	262×	50.92
Chr09	2,451,450	262×	50.80
Chr10	1,863,307	264×	50.92
Contig11	50,617	2,325×	29.15
Contig12	43,715	2,146×	46.00

### Repetitive Sequences

**Figure 3:**
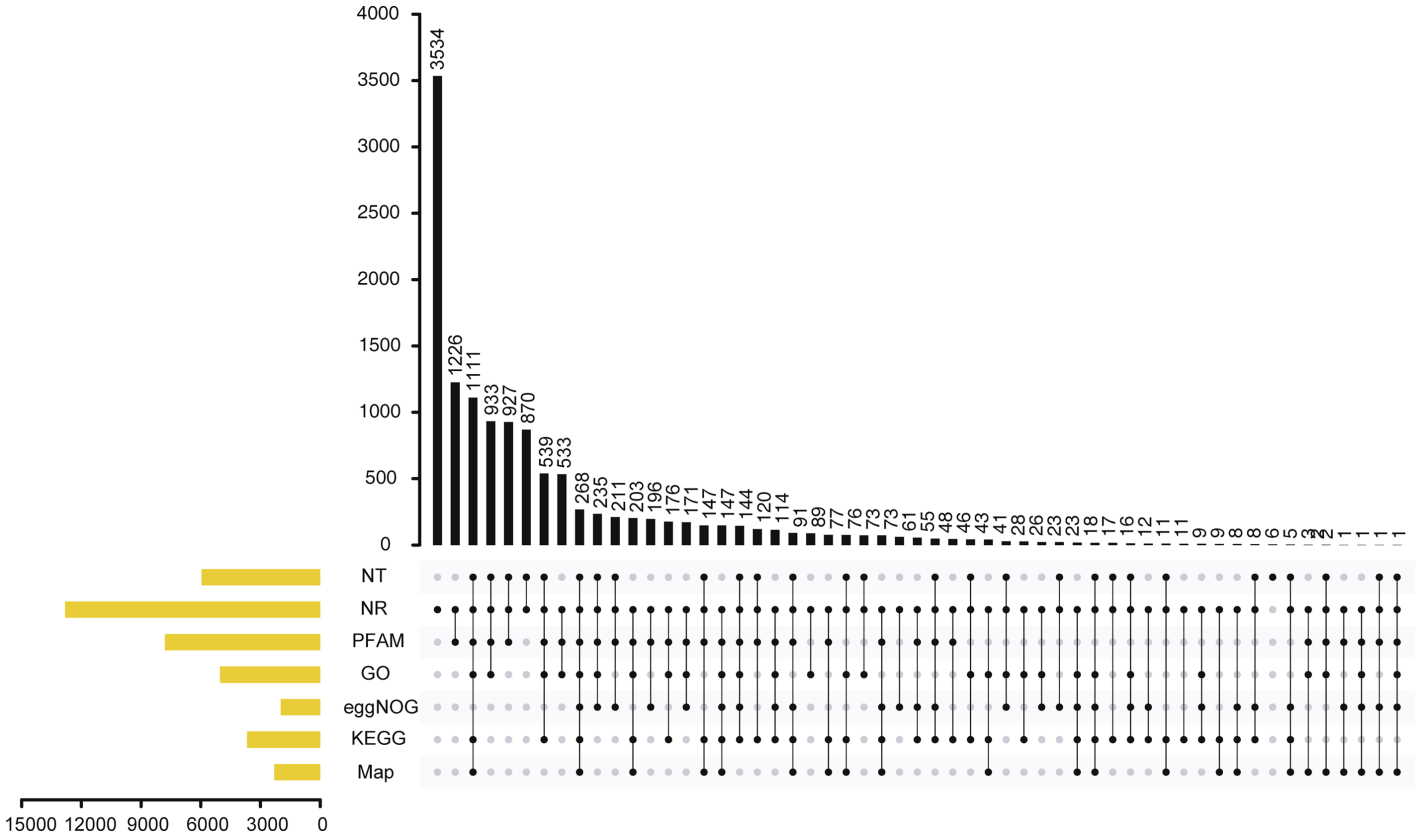
Annotation of genes in the *A. alternata* (Y784-BC03) genome as determined by Genes NR, NT, PFAM, eggNOG, GO, KEGG, and Map database analyses.

**Figure 4:**
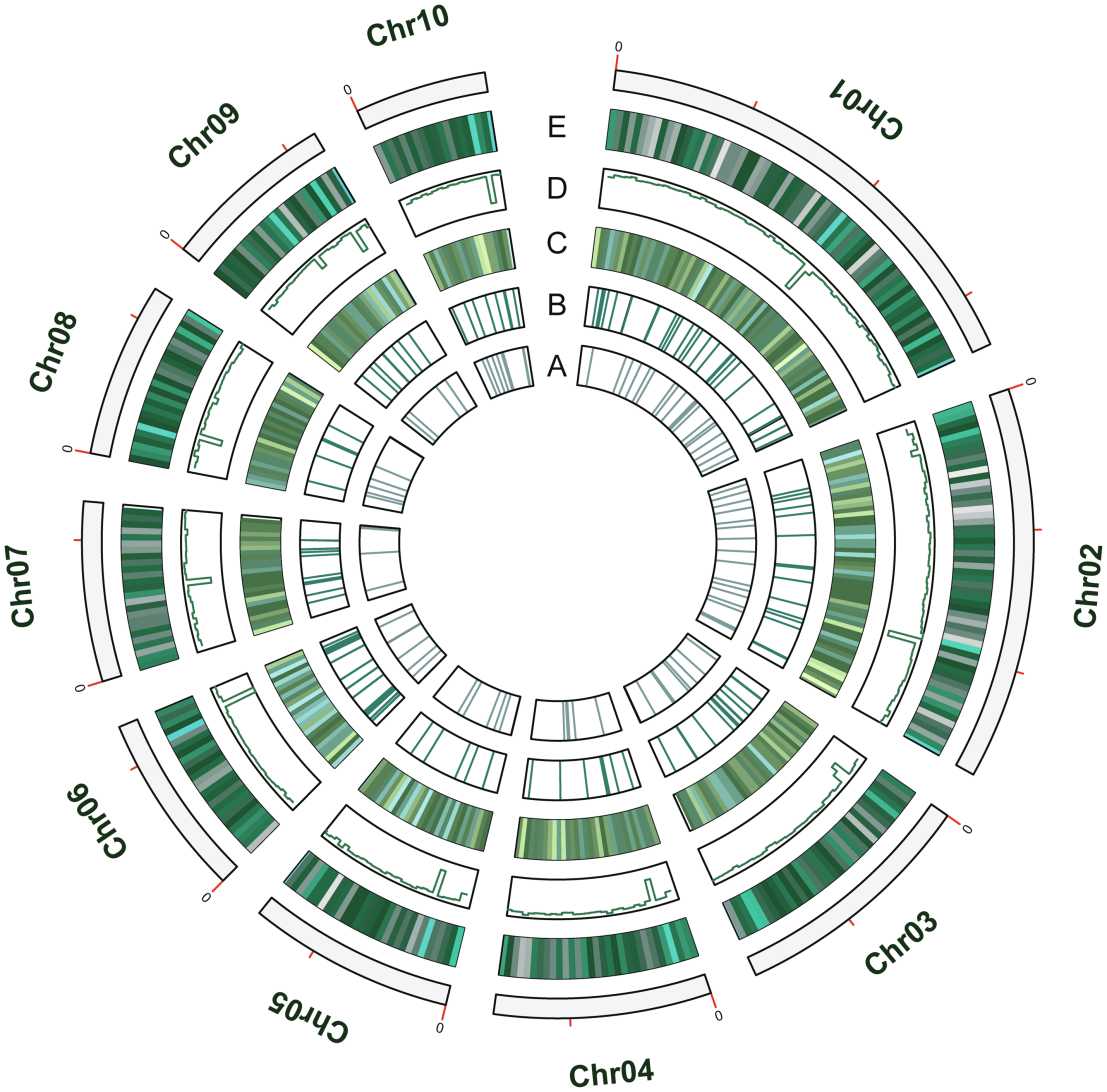
The circos diagram of the *A. alternata* (Y784-BC03) genome. Y784-BC03. **(A–E)** represent ncRNA, tRNA, toxins, GC content, and Genes, respectively.

A total of 1.7Mb of repetitive sequences were identified in *A. alternata* (Y784-BC03) and accounted for 5.27% of the genome assembly. The predicted repetitive sequences within the genome were RepeatMasker 1.03%, Protein Mask 3.01%, Denovo 2.20%, and Trf 0.28% (Supplementary Table S2). Repetitive sequences containing transposon elements, such as DNA transposon, comprised 0.56%, long interspersed nuclear elements (Line) comprised 0.13%, and barely short interspersed nuclear elements (Sine) and long terminal repeated comprised 2.88% (Supplementary Table S3). A total of 12,835 protein-coding genes were predicted with an average gene length, average coding sequence (cds) length, average exon length, and average intron length of 1657.71bp, 1477.85bp, 542.29bp, and 105.25bp, respectively (Figure 3; Supplementary Tables S4 and S5). BUSCO analysis of the predicted 99% completeness. Among the total of 290 BUSCO groups, the assembly contained 287 complete and single-copy BUSCOs (S), no duplicated BUSCOs, 1 fragmented BUSCO, and 2 missing BUSCO orthologs (Table 2). Regarding noncoding RNA, the following was predicted; 2 miRNAs, 142 tRNAs, 101 rRNAs, and 12 snRNAs (Supplementary Table S6). The assembled chromosomes, predicted genes, GC Content, tRNA, and nc RNA of the Y784-BC03 genome are presented in a circos diagram (Figure 4).

**Table 2:** Summary of BUSCO analysis of genes selected from the *A. alternata* (Y784-BC03) genome.

C:99.0% [S:99.0%, D:0.0%], F:0.3%, M:0.7%, n:290
287	Complete BUSCOs (C)
287	Complete and single-copy BUSCOs (S)
0	Complete and duplicated BUSCOs (D)
1	Fragmented BUSCOs (F)
2	Missing BUSCOs (M)
290	Total BUSCO groups searched

### Phylogenetic and Collinearity Analysis

**Figure 5:**
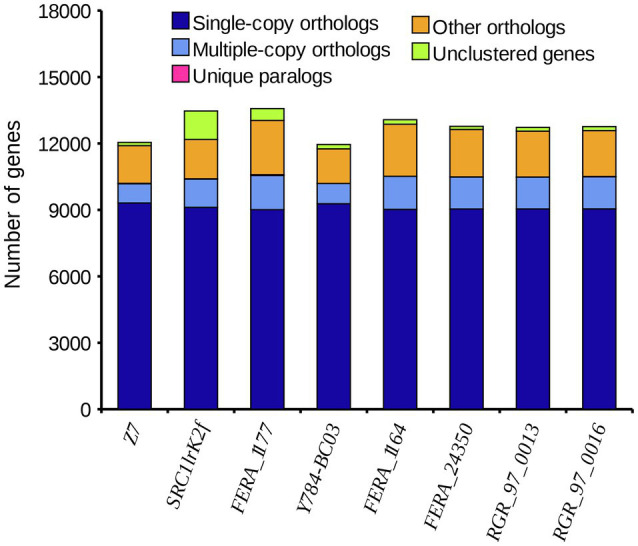
The distribution of different categories of genes in different species of *Alternaria*. The horizontal and vertical axis represent the species and the number of genes, respectively. Single-copy orthologs represent a single-copy genes that are present in all species. Multiple-copy orthologs are multi-copy genes that are present in all species. Unique paralogs are genes that are only present in the specified species. Other orthologs refer to the number of other gene families.

**Figure 6:**
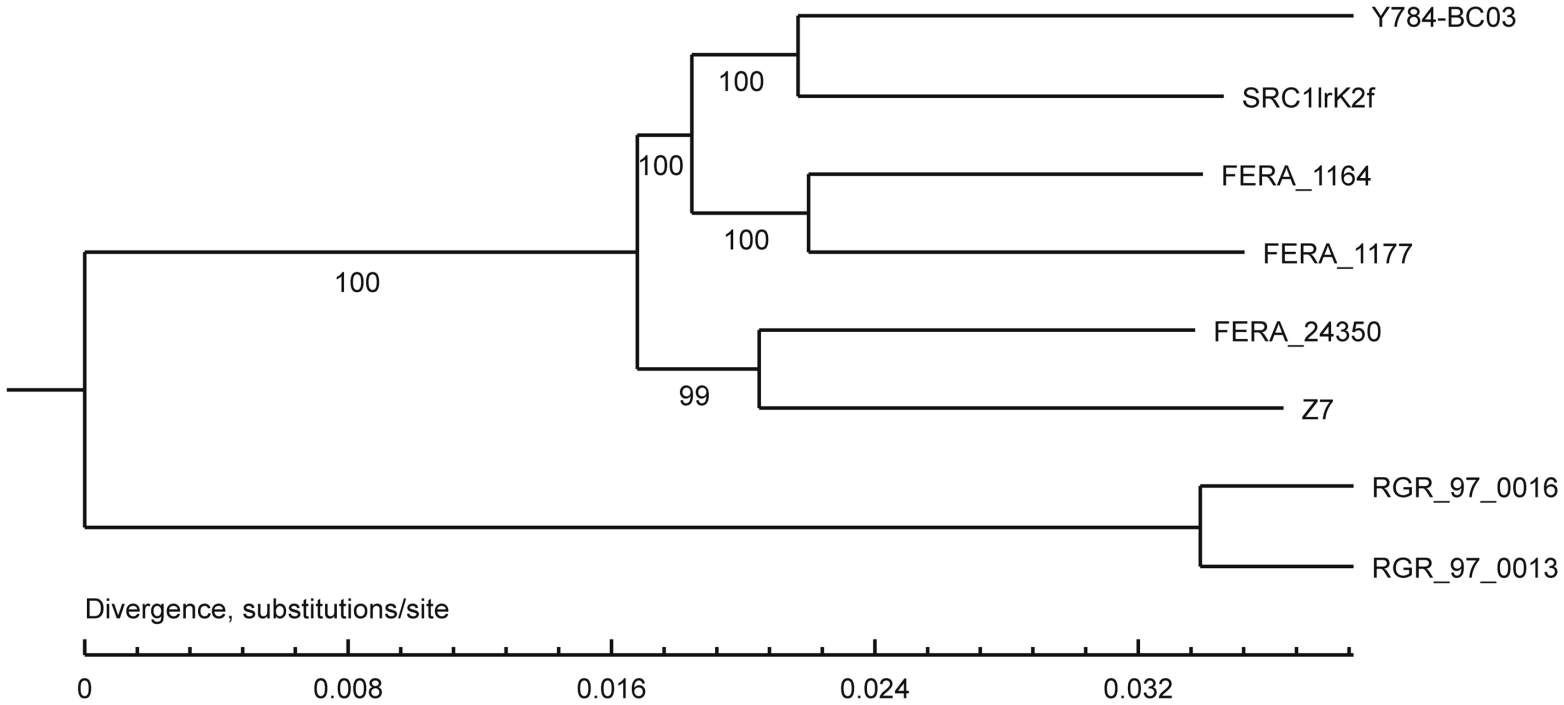
Phylogenetic analysis of *A. alternata* (Y784-BC03). The neighbor-joining tree was built with RAxML based on maximum likelihood method. Bootstrap values >50% were shown at the nodes. The scale bar represents a genetic distance of 0.1. SRC1lrK2f represents *A. alternata* isolated from effluent of coal mine drainage; Z7 represents *A. alternata* isolated from tangerine. FERA 116 4 and FERA 1177 represented *A. tenuissima* isolated from apple. FERA 24350, RGR 97.0013, and RGR 97.0016 represent non-pathotypes of *A. arborescens*.

A comparison of the *A. alternata* (Y784-BC03) genome assembly with the genome assembly of seven other *Alternaria* species revealed a total of 13,142 gene families, 9,682 of which were shared among all eight species, including 8,837 single-copy orthologous genes (Figure 5). Collectively, 11,757 genes (98.4%) of *A. alternata* (Y784-BC03) clustered into 11,231 gene families, including 1 unique gene family (Supplementary Table S1). Conserved single-copy genes were used to develop a multiple-gene tree. The phylogenetic analysis indicted that Y784-B03 was most closely related to *A. alternata* SRC1lrK2f (GCA_001642055.1; Figure 6). Therefore, the fungal strain Y784-B03 was confirmed to be *A. alternata* based on our genome analysis and multigene alignment.

### GO and KEGG Annotation

**Figure 7:**
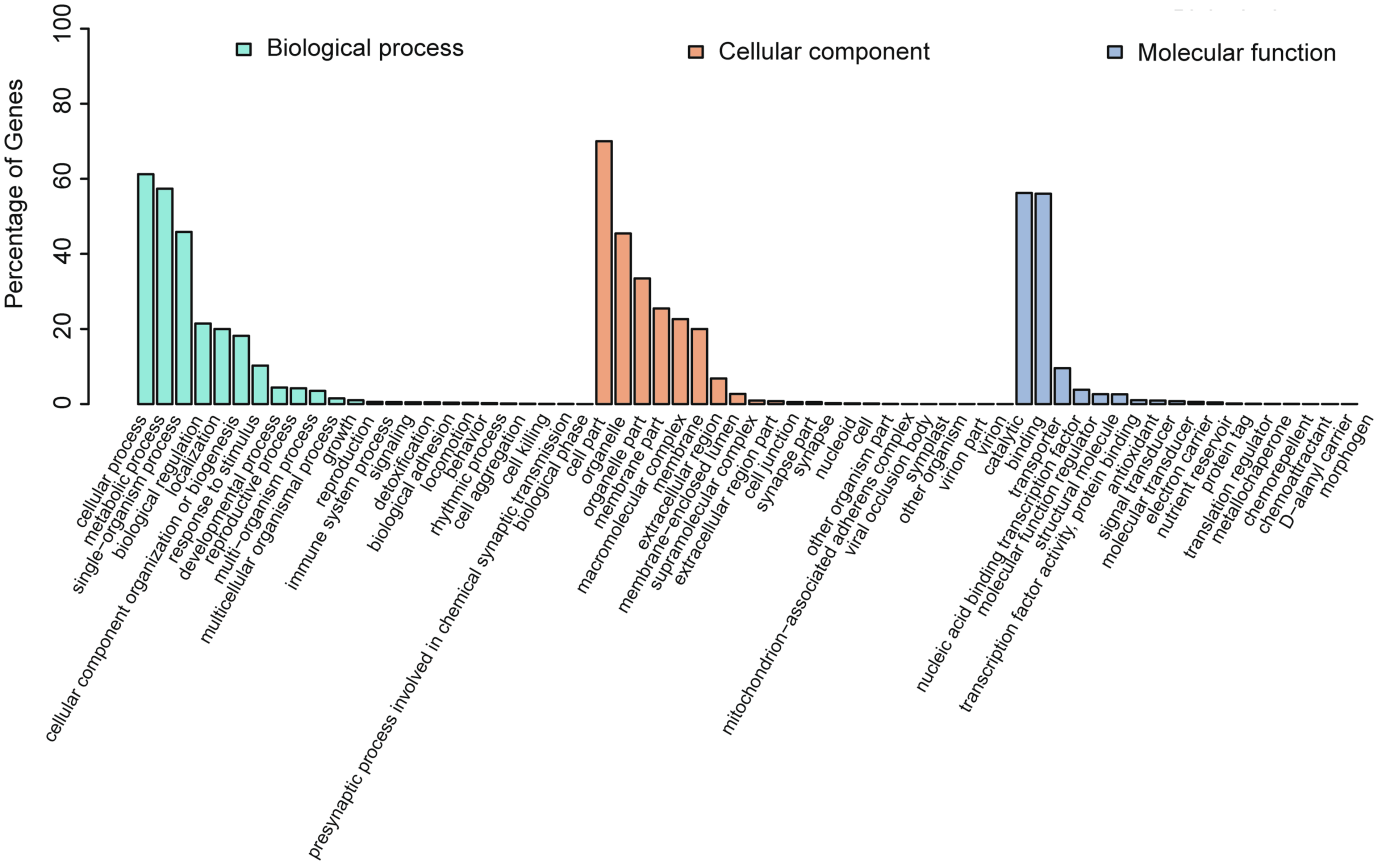
GO annotation of the predicted genes in *A. alternata* (Y784-BC03). The abscissa is the GO classification, and left ordinate is the percentage of the number of genes. This figure reveals the gene enrichment of the differentially expressed upregulated and downregulated genes. The column colors represent biological_process (green), cellular_component (red), and molecular_function (blue).

GO annotations were used to provide functional insight into the predicted genes and were categorized in the three primary GO categories of biological process, cellular component, and molecular function. Predicted genes annotated in cellular process, metabolic process, and single-organism process were abundant within biological process. Predicted genes annotated as cell part, organelle, and organelle part were abundant within cellular component (Figure 7; Supplementary Table S7), and predicted genes annotated as catalytic and binding were in the most abundant within molecular function.

**Figure 8:**
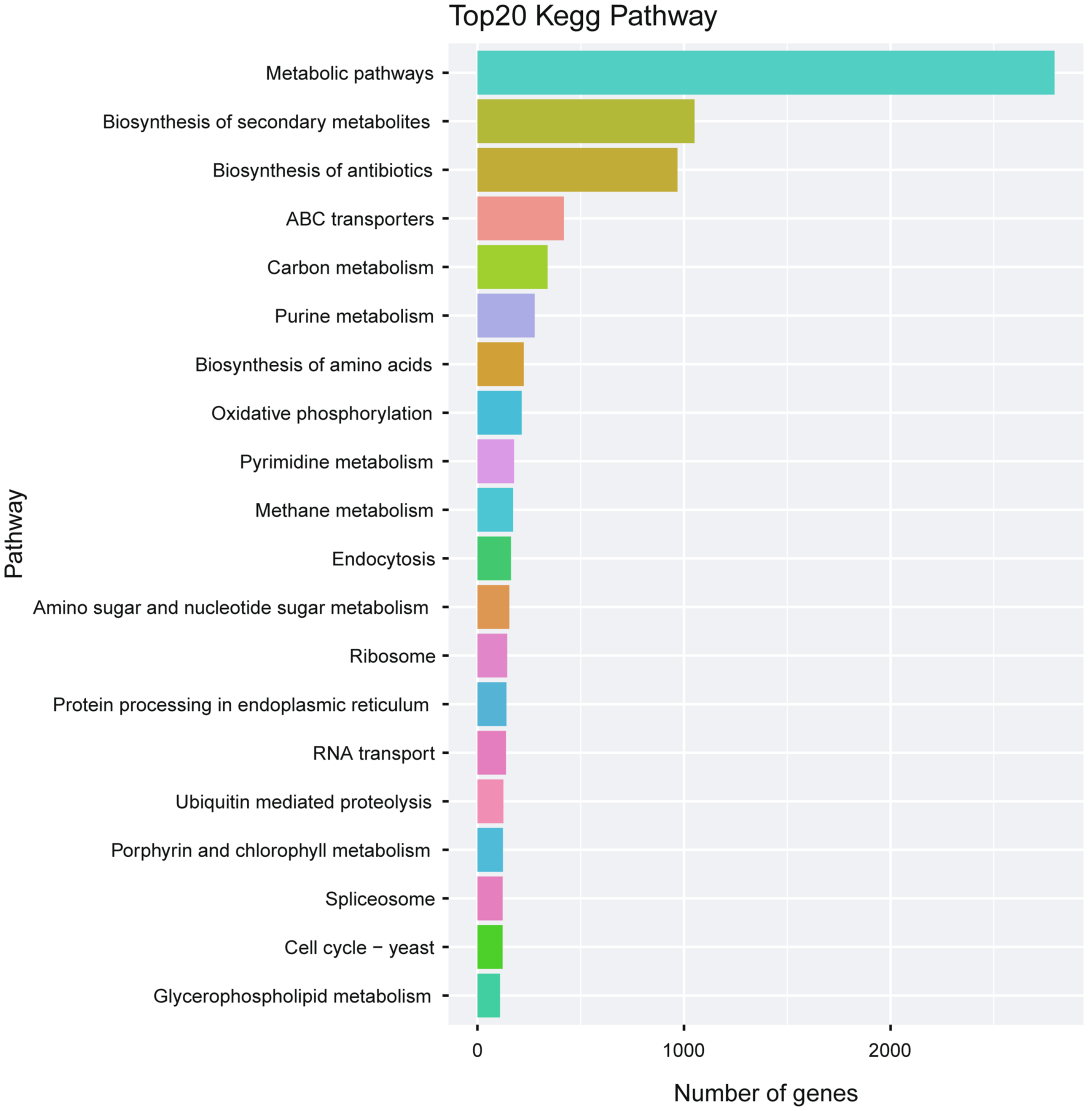
Kegg pathway analysis of the predicted genes in *A. alternata* (Y784-BC03). The Kegg bar chart indicates the top 20 pathways exhibiting gene enrichment, the horizontal axis is the number of genes, and the vertical axis is the pathway.

KEGG analysis of the predicted genes revealed an abundant number of metabolic pathways. In particular, many of the predicted genes were associated with biosynthesis of secondary metabolites and biosynthesis of antibiotics (Figure 8; Supplementary Table S7).

### Virulence Factors Predicted

A total of 2,180 genes related to virulence factors were also identified in the sequenced genome of *A. alternata* (Y784-B03; Supplementary Table S8). Fungal pathogens of plants utilize several virulence factors to ensure infection of host tissues, including effectors, secondary metabolites, and small RNA molecules (Rodriguez-Moreno et al., 2018). Secondary metabolites produced by filamentous fungi play an especially important role in fungal pathogenicity. Host-specific and general mycotoxins are generally produced by fungi as secondary metabolites, and several were identified in the *A. alternata* (Y784-B03) genome.

### Secondary Metabolites

#### Host-Specific Toxins

*Alternaria alternata* produces several host-specific toxins, including AM-toxin, AC-toxin, Ak-toxin, AF-toxins, AT-toxin, and AL-toxin (Nishimura and Kohmoto, 1983; Tsuge et al., 2013). New host-specific mycotoxins produced by *A. alternata* have also been recently reported, including AAL-toxin (Tsuge et al., 2016), ACT-toxin, ACR(L)-toxin, Maculosin toxin, Destuxin A, B, AS-toxin I, AB-toxin, HC-toxin, ABR-toxin, and AP-toxin (Meena and Samal, 2019). Genes predicted to encode ACR-toxin biosynthesis hydroxylase, AK-toxin biosynthesis protein 7, and putative HC-toxin efflux carrier TOXA, and HC-toxin bZIP transcription factor were identified in our analysis of the genome of *A. alternata* (Y784-B03; Supplementary Table S9).

#### General Toxins

General, non-host-specific, mycotoxins, such as cercosporin, dothistromin, and versicolorin B, are important virulence factors. Cercosporin is an important pathogenicity factor in *Cercospora* disease. Cercosporin was originally isolated from *Cercospora kikuchii*, the causal agent of purple stain disease in soybean (Gunasinghe et al., 2016). Cercosporin is also produced by other fungal pathogens. For instance, cercosporin produced by *Elsinoe fawcettii* plays a key role in citrus scab in numerous citrus species (Jeffress et al., 2020). Studies have also documented that cercosporin is a pathogenicity factor in white leaf spot disease in the Brassicaceae caused by *Pseudocercosporella capsellae* (Gunasinghe et al., 2016). Recently, a total of 12 cercosporin toxin biosynthesis (CTB) genes were identified and a cercosporin biosynthesis pathway was postulated. The CTB pathway may involve the following steps. CTB1, involved in non-reducing polyketide synthase (NR-PKS), is part of the first step for cercosporin production. CTB3, a predicted as O-methyltransferase/FAD-dependent monooxygenase, is involved in the synthesis of cercoquinone. Subsequently, cercosporin intermediate products are progressively synthesized by CTB2, CTB6, CTB11, and CTB12. The next step involves the synthesis of a methylenedioxy bridge by CTB5, CTB7, CTB9, and CTB10 (Newman and Townsend, 2016; Rangel et al., 2020). Our analysis of the *A. alternata* (Y784-B03) genome identified the following cersosporin biosynthesis-related genes: O-methyltransferase, *CTB2*; dual O-methyltransferase/FAD-dependent monooxygenase, *CTB3*; Cercosporin major facilitator superfamily (MFS) transporter, *CTB4*; FAD-dependent monooxygenase, *CTB5*; Ketoreductase, *CTB6*; Monooxygenase, *CTB7*; Hydroxylase/desaturase, *CTB9*; and Fasciclin-like arabinogalactan protein, *CTB11* (Supplementary Table S9).

Aflatoxins are secondary metabolites that function as mycotoxins and are harmful to human health. They can contaminate food products, such as wines, baby foods, and fruit juices, and stored grain crops. The biosynthetic pathway for aflatoxin production involves the synthesis of 15 structurally defined intermediates and 30 genes, as well as transcriptional regulatory factors. Versicolorin B is an important intermediate in the afla-toxin biosynthetic pathway (Yu et al., 2002; Yu and Ehrlich, 2011; Roze et al., 2013). *AflA*, *AflB*, *AflC*, *HypC*, *AflF*, *AflE*, *AflD*, *AflG*, *AflH*, *AflI*, *AflV*, *AflW*, *AflJ*, *AflK*, and *AflL* are genes that are in the synthesis of Versicolorin B from acetate. Notably, genes involved in the biosynthesis of dothistromin, such as *HexA*, *PksA*, *HypC*, *NorB*, *AvfA*, *CypA*, *MoxY*, and *VbsA*, are orthologs of *AflA*, *AflB*, *HypC*, *AflF*, *AflI*, *AflV*, *AflW*, and *AflK* (Schwelm and Bradshaw, 2010). Therefore, the structure of dothistromin is similar to versicolorin B (Bradshaw and Zhang, 2006). In our results, genes predicted to encode dothistromin biosynthesis protein NorB, dothistromin biosynthesis protein CypX, dothistromin biosynthesis protein A (VER-1), dothistromin biosynthesis peroxidase DotB, and efflux pump DotC, and two transcription factors (esterase 1 and norsolorinic acid ketoreductase nor1) were identified in the *A. alternata* (Y784-BC03) genome. The gene predicted to encode versicolorin B synthase (*vbs*) that catalyzes versiconal to versicolorin B, which is subsequently catalyzed to versicolorin A by *AflL*, was also identified in the *A. alternata* (Y784-BC03) genome (Supplementary Table S9).

### Key Enzymes and Regulators

The mycotoxin family described above include ACR-toxins, cercosporin, dothistromin, and versicolorin B, all of which are polyketide mycotoxins. *ACRTS1* and *ACRTS2* are function in ACR-toxin synthesis. *ACRTS1* encodes a putative hydroxylase and *ACRTS2* encodes a polyketide synthase (PKS; Izumi et al., 2012a,b). CTB1, a NR-PKS, is involved in the first step of cercosporin synthesis (Newman and Townsend, 2016; Rangel et al., 2020). PKS is involved in the second step of the synthesis of both dothistromin and versicolorin B (Schwelm and Bradshaw, 2010). Notably, genes predicted to encode polyketide synthase-nonribosomal peptide synthase and PKS were identified in the *A. alternata* (Y784-BC03) genome (Supplementary Table S9).

Plant pathogenic fungi possess a large number of cytochrome P450 monooxygenase (CYPs) genes. CYPs in fungi are involved in the synthesis of primary and second metabolites, such as aflatoxin, AK-toxin, AF-toxin, botridial, depudecin, dothistromin, ergot alkaloid, fumonisin, HC-toxin, ochratoxin, paxilline, PR-toxin, sterigmatocystin, and trichothecene (Shin et al., 2018). In addition to CYPs, CTB3, CypX, and MoxY are also involved in the synthesis of cercosporin, dothistromin, and versicolorin B. Genes predicted to encode CYPs involved in toxin biosynthesis, such as cytochrome P450 monooxygenase, trichothecene biosynthesis protein 11 (TRI1), TRI4, TRI11, NADPH cytochrome P450 monooxygenase, P450 monooxygenase, and cytochrome P450 monooxygenase were all identified in the *A. alternata* (Y784-BC03) genome (Supplementary Table S9). Notably, some CYPs (toxin biosynthesis cytochrome P450 monooxygenase, Cytochrome P450 monooxygenase TRI1, NADPH cytochrome P450 monooxygenase and P450 monooxygenase and cytochrome P450 monooxygenase) may have other functions in pathogenic fungi in addition to mycotoxin production (Shin et al., 2018).

A zinc-finger transcription factor and Zn(II)Cys6 are the two important regulators of mycotoxin synthesis. For example, AK-toxins biosynthesis gene (*AKTR*) encodes a transcriptional regulator that is a member of the Zn(II)2Cys6 family in fungi, and positively regulates AK-toxin synthesis in *A. alternata*, a Japanese pear pathotype of *A. alternata* (Meena and Samal, 2019). *CTB8* encodes a Zn(II)Cys6 transcription factor responsible for the regulation of the *CTB* cluster (Chen et al., 2007). Additionally, *aflR* encodes a zinc-finger DNA-binding protein that serves as a transcription factor regulating aflatoxin synthesis (Yu et al., 2002; Yu and Ehrlich, 2011; Roze et al., 2013). A zinc-finger transcription factor and Zn(II)Cys6 are also positive regulators of dothistromin synthesis (Bradshaw et al., 2019). Genes predicted to encode the zinc-finger and Zn(II)2Cys6 transcription factors were identified in the *A. alternata* (Y784-BC03) genome (Supplementary Table S9).

### Cell-Wall-Degrading Enzymes

Some fungal pathogens utilize cell-wall-degrading enzymes (CWDEs) to penetrate host tissues where they to degrade host cell walls primarily composed of carbohydrates. Pectate lyase is a common CWDEs previously demonstrated to play a key role in the infection of plants by *Colletotrichum lindemuthianum* (Cnossen-Fassoni et al., 2013). A gene encoding a codededpecate lyase was identified in the *A. alternata* (Y784-BC03) genome (Supplementary Table S9).

### Other Secreted Virulence Factors

In addition to CWDEs and mycotoxins, several secreted proteins were also identified in the *A. alternata* (Y784-BC03) genome, including MFS proteins, ABC transporter proteins, and global regulatory factors, such as ankyrin repeat and serine/threonine-protein kinase (Supplementary Table S9). MFS are not only responsible for the transport of a broad spectrum of substrates (Li et al., 2017), but also the ability to provide resistance to toxins that are for fungi and they also influence pathogenicity (Del Sorbo et al., 2000), acting as secondary transporters. In our results, genes encoding a putative CTB4, a cercosporin MFS transporter, and DotC encoding an efflux pump *dotC* involved in cercosporin and dothistromin synthesis and transport were identified in the *A. alternata* (Y784-BC03) genome (Supplementary Table S9). Previous studies, utilizing mutant strains, have demonstrated that an MFS transporter is an important virulence factor in *Botrytis cinerea* and *Penicillium digitatum* during the infection process (Vela-Corcía et al., 2019; de Ramón-Carbonell and Sánchez-Torres, 2021). MFS transporters have been demonstrated to be responsible for the export of fungal mycotoxin metabolites (Khaldi et al., 2010). MFS transporters and ABC transporter proteins are also involved in multidrug resistance in fungi (Gulshan and Moye-Rowley, 2007; Muñoz et al., 2018). Genes encoding MFS transporters (MFS toxin efflux pump, putative MFS allantoate transporter, and MFS transporter) and ABC transporters were identified in the *A. alternata* (Y784-BC03) genome.

The ankyrin repeat sequence comprises a sequence of conserved 33 amino acid repeated modules that mediate protein–protein interaction. APSES (Asm1p, Phd1p, Sok2p, Efg1p and StuAp) comprise a family of transcription factors unique to fungi. Ankyrin repeats are present as a domain in APSES (Zhao et al., 2015). Proteins with ankyrin repeats function in cell cycle regulation, mitochondrial enzymes, cytoskeleton interactions, signal transduction, and stress resistance (Kubicek et al., 2019).

Protein phosphorylation and dephosphorylation are essential regulatory process that occur in the metabolism of bacteria and eukaryotes. Serine/threonine-protein kinases comprise virtually all of the protein kinases present in filamentous fungi, including cAMP-dependent kinases (PKA), protein kinase type C (PKCs), mitogen-activated kinases (MAP), p21-activated kinases (PAK), and other protein kinases (Dickman and Yarden, 1999). Protein kinases have been reported to be involved in adaption and virulence in bacteria (Pan et al., 2017), the formation of fungal appressoria and ascospore proliferation (Jiang et al., 2018), and host penetration by appressoria (Zhao and Xu, 2007).

Fatty acid synthase is an important enzyme in the first step in the first step of aflatoxin synthesis (Yu et al., 2002). Additionally, aft families, ribosomal protein, non-ribosomal peptide synthetase, and *Alternaria* spp.-associated virulence factors, such as trichothecene, play an important role in mycotoxin production in fungi (Gil-Serna et al., 2020; Supplementary Table S9). Genes predicted to encode killer toxins that have the ability to inhibit filamentous fungi (Mannazzu et al., 2019) were also detected in the *A. alternata* (Y784-BC03) genome.

### Virulence Gene Clusters

**Figure 9:**
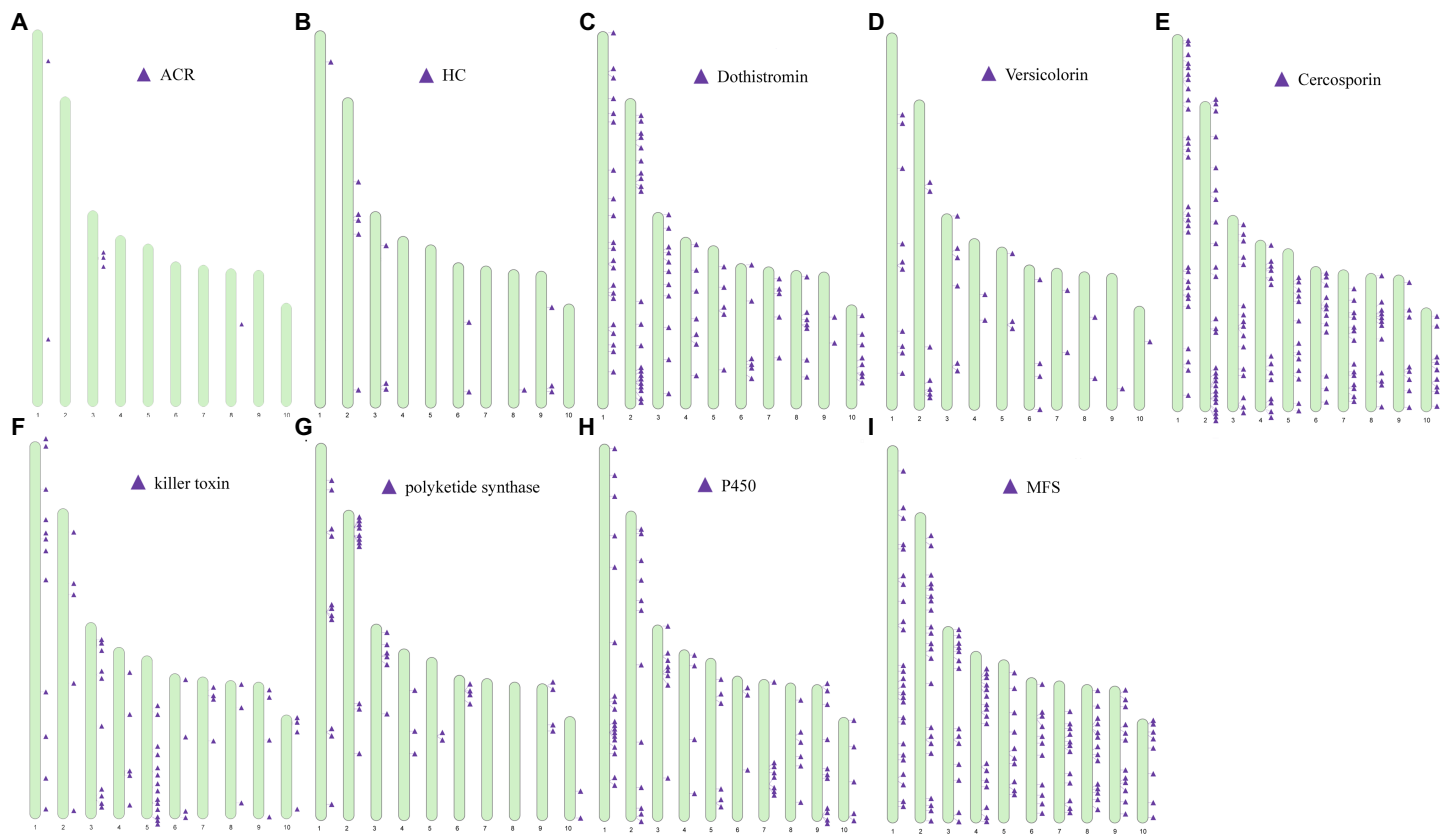
Distribution and location of toxin gene and regulator [P450s and major facilitator superfamily (MFS)] clusters on the ten chromosomes identified in *A. alternata* (Y784-BC03). **(A–I)** represent predicted toxins (ACR, HC, Dothistromin, Versicolorin, Cercosporin, killer toxin) and regulators (polyketide synthase, P450 and MFS), respectively.

Genes associated with the biosynthesis, transport, and transcriptional regulation of secondary metabolite virulence factors are generally organized in gene clusters in fungi (Khaldi et al., 2010). For example, secondary metabolite clusters are present in the genome of *Sclerotinia sclerotiorum* and *Botrytis cinerea* (Amselem et al., 2011) and *Penicillium* species (Nielsen et al., 2017). After locating the categorized virulence factors on chromosomes, we found that virulence factors were partially arranged in clusters on a chromosome (Figure 9). For example, genes predicted to be involved in the biosynthesis of ACR-toxin were clustered on ch3 (Figure 9A) and the chromosomal location of HC-toxin efflux carrier TOXA and HC-toxin bZIP transcription are indicated in Figure 9B. Cluster of genes predicted to encode proteins involved in the synthesis of dothistromin and cercosporin were distributed on all ten chromosomes (Figures 9C–E). Predicted gene clusters associated with the regulation of the biosynthesis and export of mycotoxins were also distributed on all chromosomes. PKS enzymes are enzymes involved in the synthesis of dothistromin and cercosporin precursors (Yu et al., 2002). PKS gene clusters were distributed on chr1, ch2, chr3, chr4, chr5, chr6, chr9, and chr10 (Figure 9G) but were not identified on chr1 and ch2. Predicted P450 monooxygenase and versicolorin gene clusters in aflatoxin and dothistromin biosynthesis were randomly distributed in the *A. alternata* (Y784-BC03) genome (Figure 9H). Gene clusters predicted to encode MFS transporters were also randomly distributed in the *A. alternata* (Y784-BC03) genome (Figure 9I). Gene clusters predicted to encode killer toxin were also present in the *A. alternata* (Y784-BC03) genome (Figure 9F).

## Discussion

*Alternaria alternata* is an important plant pathogen globally and can also cause disease in animals/humans due to the potential harm of toxin produced (Nishimura and Kohmoto, 1983; Meena and Samal, 2019). *A. alternata* (Y784-BC03) isolated from kiwifruits was capable of infecting kiwifruit when the concentration of pathogens were at least 10^4^ spores/ml, resulting in black spot on kiwifruits (Figure 2A). The hyphae of *A. alternata* (Y784-BC03) readily proliferated in kiwifruit during disease development (Figure 2B). Collectively, our results demonstrated that *A. alternata* (Y784-BC03) is a causal agent of black spot on kiwifruit.

Previous studies reported that black spot infections of aerial plant parts caused by *Alternaria* species involved host-specific toxins, such as AF-toxin and AK-toxin in Japanese pear, AM-toxin in apple, and ACR-toxin in rough lemon (Nishimura and Kohmoto, 1983; Meena and Samal, 2019). The analysis of virulence factors in the *A. alternata* (Y784-BC03) genome revealed the presence of genes predicted to encode genes involved in the biosynthesis of HSTs. These included ACR-toxin biosynthesis hydroxylase, a gene responsible for the completion of the ring structure of ACR-toxin (Izumi et al., 2012b), *AKT7* (a cytochrome P450 monooxygenase), which is involved in AK-toxin biosynthesis (Takaoka et al., 2014), and *TOXA* (a putative efflux carrier) and HC-toxin bZIP transcription factors (Walton, 2006). The predicted genes (ACR-toxin biosynthesis hydroxylase, putative HC-toxin efflux carrier TOXA, HC-toxin bZIP transcription factor, and *AKT7*) that we identified in the *A. alternata* (Y784-BC03) genome may only be involved in the biosynthesis pathway or as a regulator of host-specific toxin biosynthesis. Host-specific toxins in in the genome of *A. alternata* (Y784-BC03) need to be further explored. In addition to host-specific toxins, non-host-specific HST toxins were identified in the *A. alternata* (Y784-BC03) genome, including cercosporin and dothistromin. A previous study reported that cercosporin, dothistromin, and dersicolorin play an integral role in pathogenicity. Cercosporin was reported to be a key factor in citrus scab and leaf spot disease in Brassicaceae (Gunasinghe et al., 2016; Jeffress et al., 2020). Dothistromin was reported to be a key pathogenicity factor in needle blight of pines (Chettri and Bradshaw, 2016). Our genome analysis also identified predicted genes involved in mycotoxin biosynthesis pathways, such as trichothecene, which is commonly produced in many fungal species (Gil-Serna et al., 2020). We also identified predicted genes encoding other effector molecules in the *A. alternata* (Y784-BC03) genome, such as aft family proteins, ribosomal Protein, non-ribosomal peptide synthetase, fatty acid synthase, and killer toxin.

In summary, our disease assay, genome sequencing, and genomic analysis indicated that *A. alternata* (Y784-BC03) could cause block spot of kiwifruit. The genome analysis indicated that this strain could synthesize several secondary metabolites, including general, non-host-specific toxins, such as cercosporin, dothistromin, and versicolorin B, all of which are primary toxins produced by *A. alternata* that affect plants. *A. alternata* has a wide range of hosts and causes significant levels of plant disease (Meena and Samal, 2019). Our study was conducted to provide new information that could be used to manage black spot of kiwifruit. Management strategies include cultivation technology, nutrition, and field management. We have also provided new genomic information on *A. alternata*. This investigation represents our initial research on *A. alternata* (Y784-BC03) as the causal agent of black spot on kiwifruit. Further research will be conducted to better understand the mechanisms responsible for pathogenicity in *A. alternata* (Y784-BC03).

## Data Availability Statement

The datasets presented in this study can be found in online repositories. The names of the repository/repositories and accession number(s) can be found at: https://www.ncbi.nlm.nih.gov/, PRJNA727979.

## Author Contributions

KH, JT, and ZW: conceptualization. JLa: methodology. XS: software. YZ, PX, and WW: validation. XX, XW, and QW: investigation. JLi and RM: resources. JT, XS, and ZW: data curation. KH: writing—original draft preparation. ZW and JLi: writing—review and editing. KH and JLi: funding acquisition. All authors have read and agreed to the published version of the manuscript.

## Funding

This study was supported by grants from the Scientific Research Fund of Chongqing Technology Innovation and Application Demonstration Special Social and People’s Livelihood Key R&D Project (cstc2018jscx-mszdX0037), National Natural Science Foundation of China (31870675), and Chongqing Municipal Science and Technology Commission (cstc2019jscx-msxm1571).

## Conflict of Interest

XW is employed by QianTang Biotech Co. Ltd. The remaining authors declare that the research was conducted in the absence of any commercial or financial relationships that could be construed as a potential conflict of interest.

## Publisher’s Note

All claims expressed in this article are solely those of the authors and do not necessarily represent those of their affiliated organizations, or those of the publisher, the editors and the reviewers. Any product that may be evaluated in this article, or claim that may be made by its manufacturer, is not guaranteed or endorsed by the publisher.

## Footnotes

^1^ http://www.bioinformatics.babraham.ac.uk/projects/fastqc/

^2^ https://github.com/xiaochuanle/NECAT

^3^ https://www.repeatmasker.org/

^4^ http://augustus.gobics.de/

^5^ http://exon.gatech.edu/GeneMark/

^6^ https://github.com/KorfLab/SNAP

^7^ http://evidencemodeler.github.io/

^8^ ftp://ftp.ncbi.nlm.nih.gov/blast/db/FASTA/nr.gz

^9^ https://www.ncbi.nlm.nih.gov/nucleotide/

^10^ http://xfam.org/

^11^ http://eggnogdb.embl.de/

^12^ http://geneontology.org/page/go-database

^13^ http://www.genome.jp/kegg/

^14^ http://rfam.xfam.org/

^15^ http://lowelab.ucsc.edu/tRNAscan-SE/

^16^ http://orthomcl.org/orthomcl/

^17^ https://github.com/stamatak/standard-RAxML
